# Two Cases of Armour Thyroid Interference in Thyroglobulin Monitoring for Thyroid Cancer

**DOI:** 10.1155/2021/1152572

**Published:** 2021-11-17

**Authors:** Michelle Ponder, Elizabeth Lamos, Kashif Munir

**Affiliations:** ^1^University of Maryland Medical Center, 22 S Green St., RM N3E09, Baltimore, MD 21201-1554, USA; ^2^Division of Endocrinology, Diabetes, and Nutrition, University of Maryland School of Medicine, 827 Linden Avenue, 2nd Floor, Baltimore, MD 21201, USA

## Abstract

Thyroglobulin (Tg) monitoring is the biochemical standard for surveillance of recurrent differentiated thyroid cancer (DTC). Several assays are available to quantify Tg levels: immunometric assay (IMA), radioimmunoassay (RIA), and the newer liquid chromatography tandem mass spectrometry (LC-MS). It is well known that a number of entities can interfere with the accuracy of testing, and at this point in time, no one assay perfectly balances high sensitivity with low risk of interference. In this case study, we present two cases in which treatment with desiccated thyroid extract (Armour thyroid) led to a sudden elevation in Tg, which resolved when Armour thyroid was discontinued. This elevation occurred when Tg was measured with both IMA and LC-MS, which suggests direct interference from porcine Tg rather than heterophilic or thyroglobulin antibody (TgAb) interference. We suggest that patients with a history of DTC not be treated with desiccated thyroid extracts consistent with guidelines. Furthermore, more advances need to be made in the area of Tg testing to improve specificity and avoid detection of nonhuman Tg and other similar proteins.

## 1. Introduction

After successful treatment for differentiated thyroid cancer (DTC), patients are traditionally monitored for biochemical disease recurrence with serial thyroglobulin (Tg) levels [1, 2]. Tg is a protein synthesized by both healthy thyroid follicles and DTC cells. A rising serum Tg is usually the first detectable sign of DTC recurrence [3]. This noninvasive test has remained the preferred surveillance strategy for many years; however, the test is not without drawbacks. Notably, the presence of both antithyroglobulin antibodies (TgAb) and heterophilic antibodies can interfere with accuracy of the test [4]. Additionally, different assays can result in discordant results for the same sample, even in the absence of antibody interference [5, 6]. Over the years, strides have been made to mitigate these issues, including tandem monitoring of TgAb along with Tg, and attempts at standardization of assays to CRM45 [7]. Additionally, several new assays have emerged over the years in hopes of finding a new reference standard.

Tg testing is primarily performed via either the immunometric assay (IMA) or radioimmunoassay (RIA). Tg monitored via IMA is often underestimated in the presence of TgAb, as these antibodies competitively bind to Tg and prevent detection by reagent antibodies [8]. In contrast, heterophilic antibodies can result in Tg overestimation by binding directly to reagent antibodies and stimulating a response [9]. RIA in contrast is less prone to interference from TgAb and is unaffected by heterophilic antibodies; however, it is also a less sensitive test [3]. Thus, liquid chromatography tandem mass spectrometry (LC-MS) has been introduced in hopes of improving sensitivity and decreasing antibody interference. Proponents claim that this new method is free from TgAb interference, though this has been called into question by several studies [10]. Regardless, discrepancies persist among all three assays, namely, due to pre and posttranslational modification of Tg mRNA, which results in multiple different isoforms of Tg that will be detected with varying accuracy by each test [11].

After initial DTC treatment, patients are supplemented with thyroid hormone to prevent any unnecessary stimulation of thyroid tissue that could result in disease recurrence. The vast majority of patients are treated with synthetic levothyroxine (T4) per guideline recommendations. However, desiccated thyroid hormones have gained recent popularity, with individuals desiring “natural” thyroid hormone replacement, and claims that these formulations provide an advantage over synthetic T4 [12]. Additionally, patients with more longstanding disease may present on preprescribed desiccated thyroid extracts. It has not been explored whether porcine thyroid extracts, such as Armour thyroid, can affect the accuracy of Tg testing.

Here, we present two cases of patients who experienced sudden and unexpected elevations in Tg with both IMA and LC-MS testing while on suppressive therapy with Armour thyroid. In both cases, Tg levels normalized after switching to levothyroxine. This raises concerns that desiccated thyroid extract (including brands such as Armour and nature thyroid) may interfere with Tg testing.

## 2. Case Presentation

**Figure 1:**
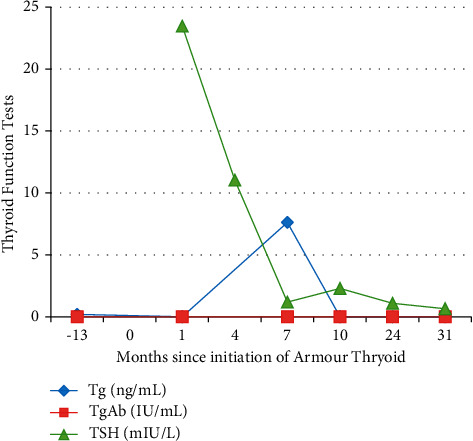
Trend of TSH and thyroglobulin results over time in case 1.

A 53-year-old woman with toxic multinodular goiter in the 1990s was treated with radioactive iodine. A new thyroid nodule developed several months after treatment and subsequently continued to grow. Fine needle aspiration was consistent with thyroid cancer. Total thyroidectomy revealed a 2 cm papillary thyroid cancer. Treatment ensued with levothyroxine 150 mcg PO daily and 100 mCi I-131 postoperatively. Initial postradioiodine whole body imaging showed uptake in the thyroid bed, with multiple subsequent diagnostic whole body scans showing no evidence of abnormal uptake. Periodic sonography and testing of Tg and Tg antibody showed no evidence of biochemical or structural disease recurrence for over 10 years. The patient requested to switch to Armour thyroid. Tg level one month later was <0.1 ng/ml and Tg antibody <1 IU/ml, performed utilizing the Quest Diagnostics Beckman Coulter IMA method (Figure 1). TSH was initially elevated at 23.47 mIU/l. Armour thyroid was adjusted to goal TSH suppression, and 7 months later, repeat testing showed Tg level of 7.62 ng/ml and Tg Ab < 1 IU/ml, performed utilizing the Quest Diagnostics Beckman Coulter IMA method. Concurrent TSH was 1.19 mIU/l on Armour thyroid 180 mg daily. The same sample was sent for LC-MS by Quest Diagnostics, which measured Tg at 9.7 ng/ml. Suspecting Armour thyroid interference with the assay, Armour thyroid was discontinued and levothyroxine therapy was initiated. Three months after restarting levothyroxine, Tg level was <0.1 ng/ml and TgAb < 1 IU/ml, performed utilizing the Quest Diagnostics Beckman Coulter IMA method. TSH was 2.29 mIU/l.

## 3. Case 2

**Figure 2:**
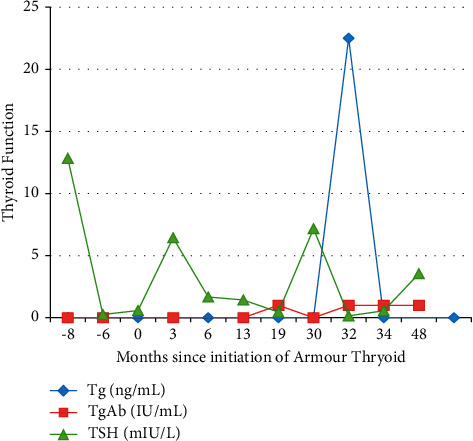
Trend of TSH and thyroglobulin results over time in case 2.

A 45-year-old woman with a long history of Graves' disease treated initially with methimazole underwent a total thyroidectomy after a fine needle aspiration of a cold nodule revealed multifocal, bilateral micropapillary thyroid cancer (largest focus 5.5 mm, classic variant). Two of 12 lymph nodes were positive for metastatic disease. Postoperative testing revealed an undetectable Tg and TgAb levels and no evidence of structural disease recurrence by thyroid ultrasound and CT of the chest, abdomen, and pelvis. The patient also preferred Armour thyroid after feeling poorly on initial levothyroxine replacement. Her Tg and TgAb remained undetectable for a year after initiation. 19 months later, Tg was <0.1 ng/ml and TgAb was 1 IU/ml performed utilizing the Quest Diagnostics Beckman Coulter IMA method while on Armour thyroid (Figure 2). Her TSH was 0.45 mIU/l at that time. 30 months after initiation, her Tg level rose to 22.5 ng/ml with Tg antibody <1 IU/ml performed utilizing the Quest Diagnostics Beckman Coulter IMA method. TSH at the time was 7.17 mIU/l. Again, the same specimen was sent for testing by LC-MS, and Tg level was 28.6 ng/ml. Armour thyroid was discontinued, and six weeks after restarting levothyroxine, Tg level was <0.1 ng/ml and TgAb was 1 IU/ml performed utilizing the Quest Diagnostics Beckman Coulter IMA method. TSH was 0.15 mIU/l.

## 4. Discussion

A sudden elevation in Tg level in any patient with a history of DTC can be alarming. However, it is always important to consider the clinical context of any lab abnormality. In the two cases described above, the sudden elevation in Tg after initiating treatment with Armour thyroid raised suspicion for interference with the test. As expected, Tg normalized after switching back to levothyroxine.

One possibility for the unexpected results may be due to antibody interference, as this is the most common reason for Tg testing inaccuracy. However, TgAb positivity generally leads to underestimation of Tg in IMA, and the opposite was observed here. Furthermore, LC-MS avoids antibody interference via use of trypsin [11], which hydrolyzes antibody complexes. Thus, if heterophilic antibodies were the cause, it is unlikely that any interference would be observed with the use of LC-MS.

A second possibility would be interference from Tg itself. Desiccated thyroid extract contains colloid thyroid tissue and thus Tg. Comparison of the structure between human and porcine Tg shows about 76% homology between the two (including 637 amino acid changes or deletions) [13]. It is unsurprising that IMA would have difficulty differentiating between the two similar molecules, as it only uses a single epitope to recognize and detect Tg [12]. However, it should be noted that a protein the size of Tg is less likely to be absorbed by the GI tract [14], and thus, direct observation of Tg in the bloodstream from Armour thyroid is unlikely.

Another possibility may involve indirect interference with the test itself, as is seen with biotin interference with thyroid function monitoring. In this case, the biotin molecule competitively competes with the binding of radiolabeled antibodies with the TSH molecule, thus resulting in a falsely low TSH level when present in high enough amounts [15]. In a similar way, an antigen present in Armour thyroid may bind with and activate the reagent, making Tg levels appear falsely elevated. Some studies suggest the possibility of larger peptide absorption, which could potentially be exacerbated by “leaky gut” that can be seen in individuals with autoimmune disorders (one case had the history of Graves' disease) [16].

At this time, the true mechanism of dissected thyroid interference with thyroglobulin monitoring is unknown. Any patients taking desiccated thyroid extracts who are suspected of having porcine Tg interference should be switched back to levothyroxine and have Tg retested shortly thereafter. To identify if the elevation in Tg is truly due to cancer recurrence (regrowth of thyroid tissue), rhTSH-stimulated Tg may be done. A physiologic rise in Tg in response to TSH stimulation would suggest that the Tg originates from the patient's own thyroid tissue, rather than from an outside source such as thyroid extract.

Guidelines for treatment for hypothyroidism recommend that patients with primary hypothyroidism be treated with levothyroxine rather than desiccated thyroid extract, as long-term outcome data are lacking [17]. However, some patients will seek the use of desiccated thyroid hormone for a number of reasons including lack of symptom improvement on traditional T4 replacement and seeking a more “natural” option. Others may unknowingly be using over-the-counter supplements containing desiccated thyroid extracts. The two cases here demonstrate desiccated thyroid hormone interference with thyroid cancer monitoring, a potential harm if associated with additional testing, imaging, and the emotional toll of an abnormal test. These cases support the current guidelines advocating for synthetic thyroxine as standard of care for thyroid hormone replacement or suppression in differentiated thyroid cancer.

## Data Availability

The data used to support the findings of this study are available from the corresponding author upon request.

## References

[1] Mazzaferri E. L., Robbins R. J., Spencer C. A. (2003). A consensus report of the role of serum thyroglobulin as a monitoring method for low-risk patients with papillary thyroid carcinoma. *Journal of Clinical Endocrinology & Metabolism*.

[2] Haugen B. R., Alexander E. K., Bible K. C. (2016). American thyroid association management guidelines for adult patients with thyroid nodules and differentiated thyroid cancer: The American thyroid association guidelines task force on thyroid nodules and differentiated thyroid cancer. *Thyroid*.

[3] Baudin E., Do Cao C., Cailleux A. F., Leboulleux S., Travagli J. P., Schlumberger M. (2003). Positive predictive value of serum thyroglobulin levels, measured during the first year of follow-up after thyroid hormone withdrawal, in thyroid cancer patients. *Journal of Clinical Endocrinology & Metabolism*.

[4] Krahn J., Dembinski T. (2009). Thyroglobulin and anti-thyroglobulin assays in thyroid cancer monitoring. *Clinical Biochemistry*.

[5] Spencer C. A., Bergoglio L. M., Kazarosyan M., Fatemi S., LoPresti J. S. (2005). Clinical Impact of thyroglobulin (tg) and Tg autoantibody method differences on the management of patients with differentiated thyroid carcinomas. *Journal of Clinical Endocrinology & Metabolism*.

[6] Spencer C., Lopresti J., Fatemi S. (2014). How sensitive (second-generation) thyroglobulin measurement is changing paradigms for monitoring patients with differentiated thyroid cancer, in the absence or presence of thyroglobulin autoantibodies. *Current Opinion in Endocrinology Diabetes and Obesity*.

[7] Spencer C. A. (2004). Challenges of serum thyroglobulin (tg) measurement in the presence of tg autoantibodies. *Journal of Clinical Endocrinology & Metabolism*.

[8] Mariotti S., Barbesino G., Caturegli P. (1995). Assay of thyroglobulin in serum with thyroglobulin autoantibodies: An unobtainable goal?. *Journal of Clinical Endocrinology & Metabolism*.

[9] Hoofnagle A. N., Roth M. Y. (2013). Improving the measurement of serum thyroglobulin with mass spectrometry. *Journal of Clinical Endocrinology & Metabolism*.

[10] Azmat U., Porter K., Senter L., Ringel M. D., Nabhan F. (2017). Thyroglobulin liquid chromatography-tandem mass spectrometry has a low sensitivity for detecting structural disease in patients with antithyroglobulin antibodies. *Thyroid*.

[11] Lopresti J. S., Hasan A. (2018). Monitoring TgAb-positive patients with differentiated thyroid cancer. *Current Opinion in Endocrine and Metabolic Research*.

[12] Hoang T. D., Olsen C. H., Mai V. Q., Clyde P. W., Shakir M. K. M. (2013). Desiccated thyroid extract compared with levothyroxine in the treatment of hypothyroidism: A randomized, double-blind, crossover study. *Journal of Clinical Endocrinology & Metabolism*.

[13] http://www.ncbi.nlm.nih.gov/tools/cobalt/cobalt.cgi.

[14] Barnes B. O., Carlson A. J., Riskin A. M. (1931). Studies on thyroglobulin. *American Journal of Physiology-Legacy Content*.

[15] Ardabilygazir A., Afshariyamchlou S., Mir D., Sachmechi I. (2018). Effect of high-dose biotin on thyroid function tests: Case report and literature review. *Cureus*.

[16] Miner-Williams W. M., Stevens B. R., Moughan P. J. (2014). Are intact peptides absorbed from the healthy gut in the adult human?. *Nutrition Research Reviews*.

[17] Jonklaas J., Bianco A. C., Bauer A. J. (2014). Guidelines for the treatment of hypothyroidism: prepared by the American thyroid association task force on thyroid hormone replacement. *Thyroid*.

